# Investigating the Prevalence of Copper and Zinc Abnormalities in Patients Pre and Post bariatric Surgery—an Australian Experience

**DOI:** 10.1007/s11695-023-06822-w

**Published:** 2023-09-29

**Authors:** Nazy Zarshenas, Linda C. Tapsell, Marijka Batterham, Elizabeth P. Neale, Michael L. Talbot

**Affiliations:** 1 School of Medical, Indigenous and Health Sciences, Faculty of Science Medicine and Health, University of Wollongong, Wollongong, NSW Australia; 2 Shore Surgical, 156-158 Pacific Highway, Greenwhich, Sydney, NSW 2065 Australia; 3 School of Mathematics and Applied Statistics, Faculty of Engineering and Information Sciences, University of Wollongong, Wollongong, NSW Australia; 4 St George and Sutherland Clinical School, St George Private Hospital, Suite 3 Level 5 1 South St, Kogarah, Sydney, NSW 2217 Australia; 5 University of New South Wales, Sydney, Australia

**Keywords:** Bariatric surgery, Nutritional abnormalities, Zinc, Copper, Multivitamin supplementation

## Abstract

**Introduction:**

Bariatric surgery predisposes patients to nutritional deficiencies. There are limited studies on zinc and copper abnormalities in this cohort.

**Purpose:**

The aim of this study was to identify the prevalence of these abnormalities in a cohort of Australian bariatric patients. Inflammatory markers, adherence to multivitamin supplementation (MVS) and the presence of gastrointestinal (GI) symptoms were also investigated.

**Material and Methods:**

Data was collected on all patients who attended a single clinic in Sydney, Australia, from August 2020 to August 2021.

**Results:**

The study cohort consisted of 231 patients (76.2% female; mean pre-operative body mass index of 43.4 ± 7.1 kg/m^2^), most of whom underwent sleeve gastrectomy (78.8%). Data were collected preoperatively and then at ≤ 6 months, 1 and > 2 years postoperatively. Prior to surgery, low levels of zinc (2.1%) and copper (0.7%) were rare, but elevated copper levels were common (16.7%) and potentially related to an elevated C-reactive protein (CRP) (47.7%). Following surgery at > 2 years, the mean total weight loss (TWL) was 33.5 ± 12.4. CRP levels improved over time. Post operatively, low zinc (2.7–3.6%) and copper (1.5%) levels were rare. Patients with low levels in zinc and copper were a higher-risk group and generally exhibited GI symptoms, despite taking MVS.

**Conclusion:**

In the initial post-operative stages and with good adherence to MVS containing copper and zinc, abnormalities may not be a concern. Patients with GI symptoms appear to be at higher risk of abnormalities; increasing awareness, thorough screening, and more comprehensive supplementation are recommended.

**Graphical Abstract:**

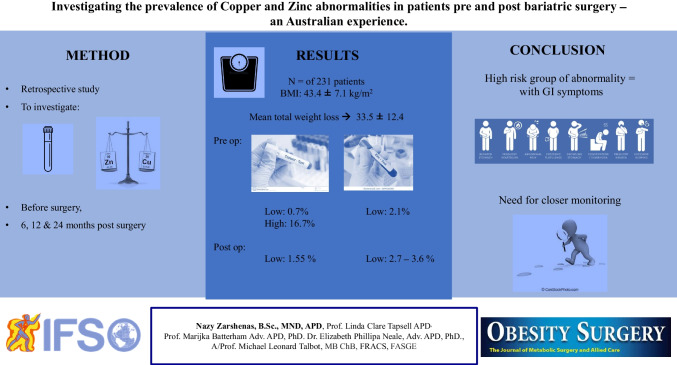

## Introduction

The durable significant weight loss, as well as the physiological benefits of metabolic and bariatric procedures, improve the medical disorders associated with the condition of obesity [1–4]. Bariatric procedures such as Roux-en-Y gastric bypass (RYGB), one anastomosis gastric bypass-mini gastric bypass (OAGB-MGB), sleeve gastrectomy (SG), biliopancreatic pancreatic diversion (BPD) and laparoscopic adjusted gastric bypass (LAGB) impact the nutritional status of patients [5, 6]. The literature identifies the most common nutritional abnormalities following bariatric surgery to be vitamins D, B_12_, folate, iron, ferritin and hyperparathyroidism [5–8]. However, there is limited data on other nutrients such as copper (Cu) and zinc (Zn), with inconsistent recommendations from bariatric guidelines on routine screening and appropriate supplementation [5–7, 9].

Copper and zinc are both essential micronutrients in metabolism. Copper is a cofactor for several essential enzymes known as cuproenzymes and is involved in energy production. It is naturally present in foods such as beef, liver, crustaceans, milk, legumes, whole grains, nuts and seeds. Copper deficiency results in haematological and neurological symptoms; commonly anaemia, leukopenia and neutropenia. Neurological symptoms, if unidentified and untreated, can result in severe and potentially irreversible complications [10–13]. Zinc is essential in pathways for growth, reproductive function, immunity as well as neuro-behavioural development. Hence, its deficiency has been shown to result in emotional disorders, poor wound healing, taste changes, glossitis and hair loss [7].

Bariatric surgery significantly reduces the intake of dietary sources for Cu and Zn. In addition, some procedures impact the absorption of these nutrients. Therefore, guidelines recommend a comprehensive daily multivitamin and mineral supplement (MVS) with at least 2 mg of Cu and 15-mg Zn in order to meet the requirements. The evidence shows that excess Zn results in upregulation of metallothionine, which has a higher affinity for Cu and, hence, prevents its release into plasma, resulting in a Zn-induced Cu abnormality [12, 13]. Therefore to prevent this, it is recommended to maintain a a ratio of 8–15 mg of Zn for each 1-mg Cu as well as monitoring levels closely [7].

Furthermore, the literature suggests that in interpreting both Cu and Zn levels, it is important to note inflammation markers such as CRP. Copper is a positive acute phase reactant [14], and hence, with the presence of inflammation and elevation of CRP, it is often elevated. In contrast to Cu, Zn is a negative phase reactant, and in condition of obesity, lower serum zinc levels as well as lower concentrations of Zn in plasma and erythrocytes have been observed. Therefore, abnormal levels of both need to be interpreted with caution as the inflammatory nature of the obesity condition, and hence, elevated CRP levels impact the serum levels of these [14].

Different recommendations for monitoring levels and for providing optimal supplementation of these trace elements can cause confusion. Additionally, there are limited comprehensive studies, especially in the Australian population, exploring Cu and Zn levels. The aim of this study was to identify the prevalence of abnormalities in both Cu and Zn in our bariatric surgical population. Inflammatory markers, other related deficiencies and adherence to supplementation were also investigated.

## Methods

The study accessed records of patients who attended a multidisciplinary private clinic in Sydney, Australia, from August 2020 to August 2021. Data included anthropometric measures, Cu, Zn and CRP levels as well as adherence to supplements and gastrointestinal symptoms. Data were collected preoperatively and then at ≤ 6 months, 1 and 2 years or more postoperatively.

All patients who attended the clinic during the study period had undergone a multidisciplinary assessment as per bariatric guidelines [5, 15] and completed routine pre- and post-operative blood tests, which were included in this study.

### Clinic Protocol

The clinic protocol incorporated routine consultation by the surgeons and an Accredited Practising Dietitian with expertise in bariatric surgery. Individualised nutritional counselling was provided, and this was based on several international bariatric guidelines [5, 6, 15, 16]. Preferably bariatric multivitamin and mineral formulas (with 0.75 to 3 mg of Cu and 15 to 28 mg of Zn) or a comprehensive MVS (with 1.8 mg of Cu and 16 mg of Zn) was recommended [5, 6, 15, 16]. Additionally, other micronutrient supplementations (vit D, calcium, B12, folate and iron) were recommended to meet the individual patients’ requirements. No additional Cu and Zn supplement was recommended unless patients had abnormal levels.

### Anthropometry

A Wedderburn scale was used for measuring weight and body mass index (BMI) (weight (kg) /height^2^ (cm)), weight loss (WL), percentage of total weight loss (%TWL) (weight loss (kg)/pre op weight × 100) and percentage of excess weight loss (%EWL) were calculated (weight loss (kg)/EW (kg) × 100).

### Biochemical Assessments

The nutritional and biochemical markers reported were Cu, Zn and CRP. In those with abnormal levels, other nutritional abnormalities were also explored. Levels of nutrients were assessed based on the standard laboratory values (please see Table 2 for normal levels).

### Adherence and Uptake of Multivitamin and Mineral Supplementation

During routine consultations, patients reported adherence to the recommended MVS was recorded at each time point.

### Gastrointestinal Symptoms

The gastrointestinal symptoms reported by patients were also recorded.

## Ethics

The data was deidentified (by a researcher who was not involved with data collection and analysis) prior to analysis. The study was approved by the University of Wollongong/Illawarra Shoalhaven Local Health District Human Research Ethics Committee (HE:2020/172), and for this type of study, formal consent was not required.

## Statistical Analysis

Descriptive statistics were expressed as mean ± standard deviation for continuous variables (anthropometry and analytical variables) and percentages for categorical data (deficiency or compliance rates). Inferential analysis was performed using IBM^®^ Statistical Package for the Social Sciences^®^ (SPSS^®^) version 27. Linear mixed models were used to compare baseline and follow-up data and Bonferroni post-hoc test to pair-wise comparisons. A *P* value < 0.05 was considered statistically significant.

## Results

### Demographics

The baseline characteristics have been described in a previously published study [17]. A total of 231 patients had a complete pre- and or post-operative Cu and Zn and were all included in the study. The majority of the procedures were done as a primary procedure (*n* = 185, 80%), with the main procedure being; SG (78.8%), followed by RYGB (15.6%) (Table 1).

**Table 1:** Patients’ characteristics

	Total
Number of patients	231
Gender ratio female/male (% F/M)	176/55 (76.2/23.8)
Age—at the time of surgery (years) (Range)	47.0 ± 11.8 (18–73)
Body weight (kg ± SD) (Range)	122.1 ± 23.6 (74.4–220.0)
BMI (kg/m^2^ ± SD) (Range)	43.4 ± 7.1 (31.0–66.5)
Excess weight (kg ± SD)	51.5 ± 19.8
Surgery types (%)	LSG: 182 (78.8%) RYGB: 36 (15.6%) OAGB-MGB: 9 (3.9%) LAGB: 3 (1.3%) Banded SG: 1 (0.4%)
Primary/revisional surgery (%)	185/46 (80/20)
Primary surgery	LAGB: 32 (70.0%) LSG: 4 (8.7%) LAGB and LSG: 8 (17.3%) GS: 1 (2.2%) Fixed band: 1 (2.2%) ESG: 1 (2.2%)

*LSG* lap sleeve gastrectomy, *RYGB* Roux-en-Y gastric bypass, *OAGB* -*MGB* one anastomosis gastric bypass - mini gastric bypass, *GS* gastric stapling, *ESG* endoscopic sleeve gastrectomy

### Anthropometric Outcomes

The changes in anthropometric measures following bariatric procedures are outlined in Table 2. Following surgery at ≥ 2 years, the total weight loss (TWL) was 28.1 ± 8.9 kg. Weight loss was significant at each time point compared to the pre-operative weight (*P* = < 0.001), stabilising at 1 year post-operative with no significant difference in measures after 1 year.

**Table 2:** Laboratory values and percentage of abnormalities at each assessment point

Biochemical marker (Normal range)	Time	*N*	Minimum	Maximum	Mean ± SD	Abnormalities *N* (%)
Zinc (10–18 μmol/L)	Pre op	142	8.50	22.0	13.4 ± 2.5	3 (2.1)
	≤ 6/12 post op	117	8.7	20.0	13.2 ± 2.3	3 (2.6)
	1 year post op	73	7.0	31.0	13.8 ± 3.6	2 (2.7)
	≥ 2 years post op	70	8.5	29.0	13.4 ± 3.1	1 (1.4)
Copper (12–22 μmol/L)	Pre op	138	11.0	43.4	20.0 ± 5.1	Low: 1 (0.7) High: 23 (16.7)
	≤ 6/12 post op	115	12.0	39.0	19.5 ± 4.7	Low: 0 (0) High: 14 (12.2)
	1 year post op	73	12.0	38.0	18.7 ± 4.6	Low: 0 (0) High: 7 (9.6)
	≥ 2 years post op	69	9.7	33.0	17.7 ± 4.1	Low: 1 (1.5) High: 6 (8.7)
CRP (0.0–0.5 mg/L)	Pre op	109	0.5	48.6	7.7 ± 7.3	52 (47.7)
	≤ 6/12 post op	83	0.4	24.0	4.5 ± 4.6	21 (25.3)
	1 year post op	48	0.4	85.5	5.5 ± 12.6	6 (12.5)
	≥ 2 years post op	62	0.4	25.7	2.5 ± 3.6	5 (8.1)

*SD* standard deviation

### Nutritional Outcomes

#### Pre-operative Nutritional Disorders

Prior to bariatric surgery, low levels of Zn were noted in 3 (2.1%) patients. They were all female, with two undergoing revisional RYGB, following their failed LAGB. One patient with other available nutrition markers also had ferritin deficiency (11 ug/L), but all other nutrition markers (B12, active B12, vitamin D), as well as CRP levels, were within normal range. It was noted that all three cases with low levels of Zn had normal Cu levels.

Low pre op Cu level was seen in one (0.7%) female patient, seeking a SG as her primary bariatric procedure. She also had anaemia (low Hb), but all her other nutrition markers (ferritin, B12, active B12, vitamin D, folate), as well as CRP, were within normal range. Elevated Cu levels, however, were present in 23 (16.7%) and elevated CRP levels in 52 (47.7%) cases. CRP levels were available in 17 out of these 23 patients, with 14 of those being elevated.

#### Post-operative Nutritional Disorders

In the period ≤ 6 months, low level of Zn was present in 3.6% patients (*n* = 3); all of which had primary procedures (*n* = 2 RYGB and *n* = 1 SG). One patient (SG) with available bloods had normal Cu and CRP; however, all her other nutrition markers (ferritin, B12, active B12, vitamin D, folate) were also below normal range. This patient reported to be taking the multivitamin supplement. However, she also reported gastrointestinal (GI) related symptoms, such as nausea and vomiting, and suffered from a significant mental health condition, making tolerance of diet and supplements problematic.

None of the *n* = 115 patients with available Cu levels showed any abnormalities at ≤ 6 months.

At 1 year post op, two patients (2.7%) exhibited Zn abnormality. Both cases were male who had undergone SG and also showed abnormalities in Fe, B12, active B12, vitamin D and elevated CRP levels. One patient reported adhering to the MVS, despite reported GI symptoms of vomiting and food intolerances. The other case had an OAGB, denied any foregut symptoms but reported malabsorptive symptoms of chronic diarrhoea/steatorrhoea. He reported adhering to a multivitamin, calcium with D, vitamin D and did not exhibit other nutritional abnormalities. However, he did have abnormally high levels of vitamin B6, and this was related to self-prescription of magnesium supplements (which included significantly high vitamin B6 levels) for management of leg cramp and pain.

No low levels of Cu were observed at 1 year. At ≥ 2 years post-operative, there was only one case with both low levels of Zn and Cu.

In this cohort of patients, high levels of Cu were more prominent than low levels. This was high pre operatively (*n* = 23) and reduced over time following surgery (*n* = 6) (Table 2).

#### Change in Mean Nutritional Levels Following Surgery

**Fig. 1:**
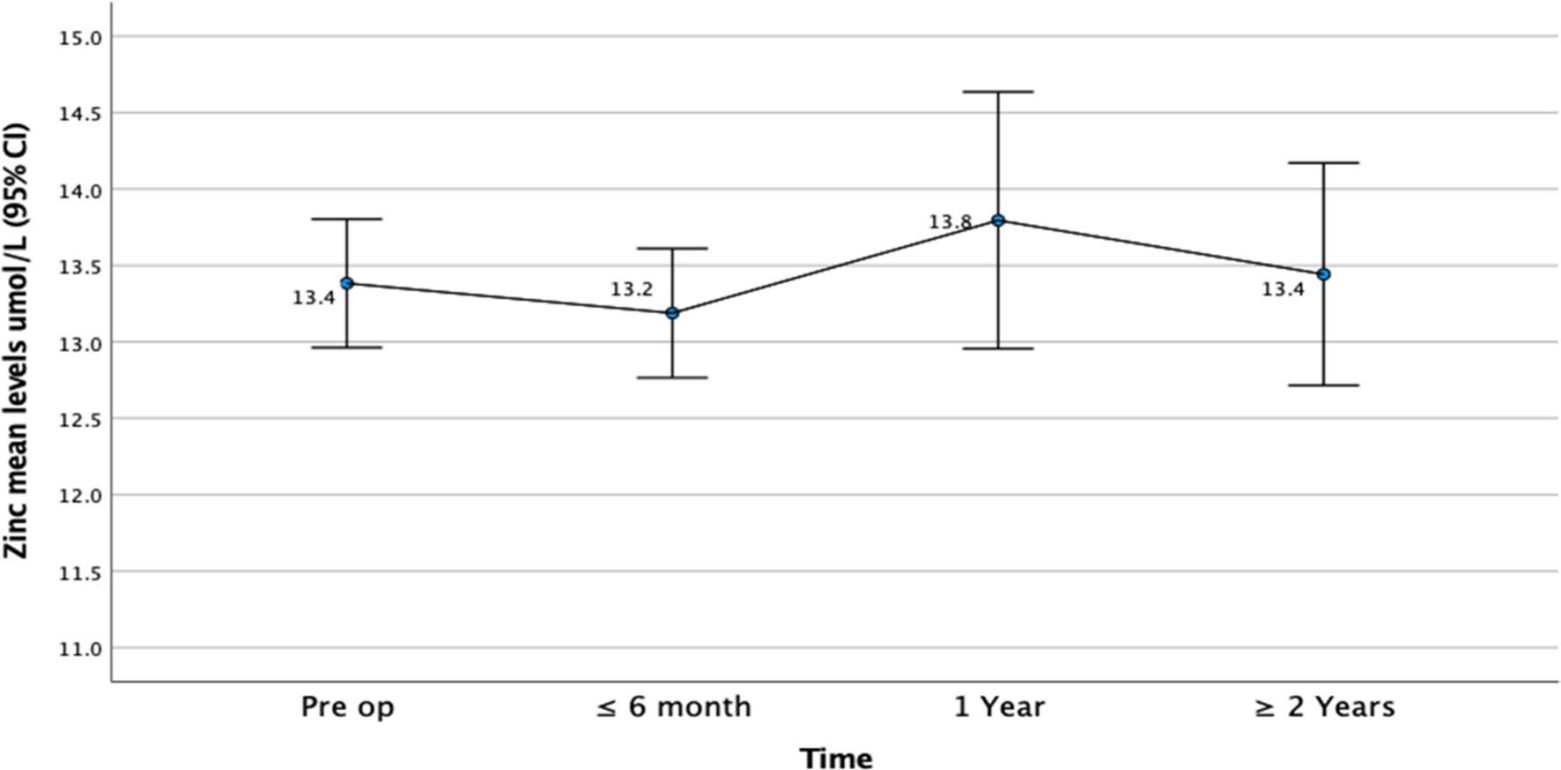
Mean zinc levels before and following bariatric surgery

**Fig. 2:**
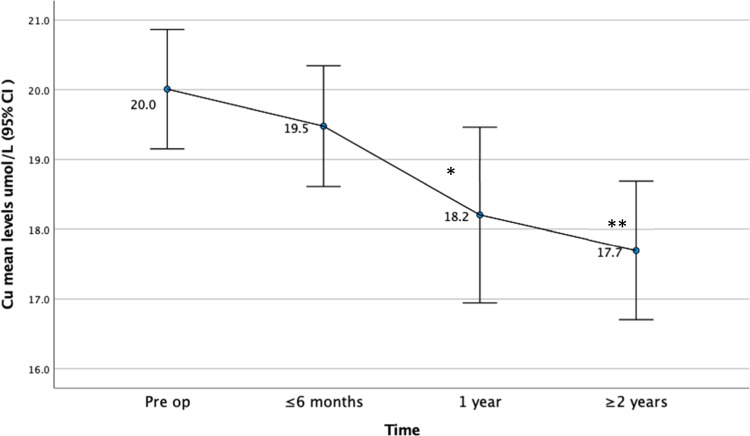
Mean Copper levels before and following bariatric surgery. *Significant compared to pre op *P* = < 0.001. **Significant compared to pre op *P* = 0.001

**Fig. 3:**
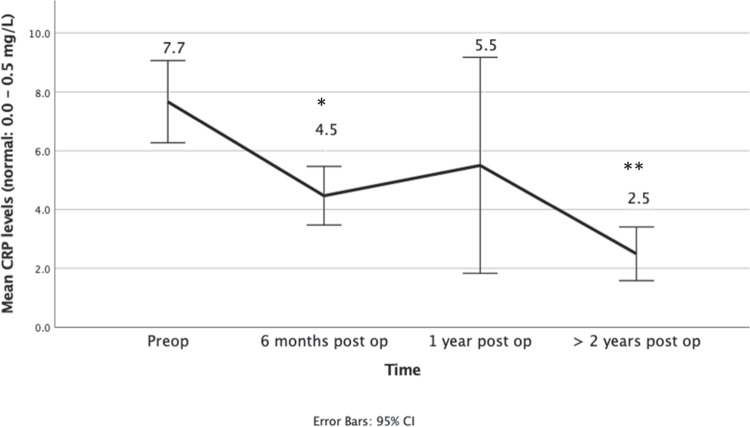
Mean CRP levels before and following bariatric surgery. *Significant compared to pre op *P* = 0.004. **Significant compared to pre op *P* = < 0.001

The mean levels of both Zn and Cu stayed within normal range at all-time points. There was no significant change in the mean Zn levels over time (Fig. 1). However, the mean levels of Cu did reduce, with a significant difference at 1 year and ≥ 2 years post-operative compared to the pre-operative values (Fig. 2)*.* CRP levels improved over time (Fig. 3).

### Adherence and Uptake of Multivitamin and Mineral Supplementation (MVS)

The recommended MVS in this cohort was a bariatric comprehensive bariatric or general ‘over-the-counter’ multivitamin which included Zn and Cu. Additional Zn and Cu were not recommended unless patients were exhibiting low levels. All patients with low levels in Zn and Cu reported to be adherent to a multivitamin and mineral supplement. The details of adherence to the MVS over time and additional recommended supplementations have been described in a previously published study [17]. However, overall adherence to the multivitamins was 90% in the first year following surgery but declined to 77% at 2 years and beyond. In this cohort, 60% of patients were taking additional calcium and vitamin D supplements with again a decline over time. Iron and vitamin B12 are generally recommended based on patients’ requirements.

### Reported Gastrointestinal (GI) Symptoms and Hospital Readmissions

In this study, food intolerances, nausea and vomiting, diarrhoea/steatorrhoea and dumping syndrome were identified to be the most commonly reported symptoms in those with low levels of Zn and Cu.

One patient with severe GI symptoms, including diarrhoea and steatorrhea, required hospital readmission and revisional surgery to correct his malabsorptive symptoms, and another patient with a very complicated medical and mental health history required multiple readmissions for intravenous therapy as well psychological support.

A more detailed description of GI symptoms and their change over time in this cohort have been described in a previously published study [17].

## Discussion

This study, consistent with others [18–22], shows that bariatric surgery results in significant weight loss (TWL: 30.9 ± 10.7), stabilising beyond 2 years following surgery. Nutritional abnormalities are a concern following bariatric surgery; however, reporting of Zn and Cu abnormalities have been limited and inconsistent [23]. Case studies do report severe and irreversible complications, justifying closer monitoring of levels of these trace elements [10]. In this cohort of patients, low levels of Zn and Cu were minimal at both pre- and post-operative. The reason for these findings may be partially related to the fact that most patients in this study had a primary bariatric procedure, mostly SG (78.7%), nearly all (90 %) of patients reported adhering to a comprehensive MVS and potentially had an adequate dietary source of Zn and Cu.

### Zinc Abnormalities

Routine screening of Zn has been recommended for patients before bariatric surgery [5]; however, reported abnormalities in Zn status remain inconsistent. The reported prevalence of Zn deficiency prior to bariatric surgery ranges from 24–28% [5, 6] to 73.9% [7, 24]. However, in our cohort, low levels of Zn were noted in only 3 cases (2.1%). Our findings are similar to Van Rutt et al., who report a 5% newly diagnosed Zn deficiency in patients following SG [25]. This is lower than the finding of others, which report a Zn deficiency level up to 15% at 1 year following surgery in a mixed cohort of bariatric patients [12]. In our study at ≤ 6 months stages following surgery, one patient exhibited Zn abnormality as well as other abnormalities in ferritin, B12, active B12, vitamin D, and folate; however, her Cu and CRP levels remained in the normal range. This patient reported to be adhering to the MVS. However, she also reported nausea and vomiting, suffered from a mental health condition, and hence may have had questionable tolerance of diet and supplementation. Most patients in our study cohort had a SG (78.8%) and reported adhering to the recommended supplementation. Hence, these may explain the difference in findings.

Overall presence of GI symptoms, non-adherence to supplementation, inadequate Zn levels in supplementation and having a more malabsorptive procedure seem to be contributing to higher Zn abnormality levels with a small number of patients developing clinical symptoms and true deficiency [26].

Zn is a negative acute phase reactant [14], and in patients with obesity, lower serum Zn levels as well as lower concentrations of Zn in plasma and erythrocytes have been observed. Thus, the levels should also be interpreted with caution, and repletion of Zn are only indicated when levels are severely low and in the presence of clinical signs and symptoms [5]. Hyperinsulinaemia is common in the bariatric surgical cohort, and it has been known to increase urinary Zn excretion [27];  in interpreting Zn levels, it too needs to be considered. In this study, hyperinsulinemia levels were not documented; however, this should be considered in future studies.

Zinc deficiency has been reported following all bariatric procedures in varying degrees and more so with the malabsorptive procedures [27]. Its deficiency has shown to result in emotional disorder, poor wound healing, taste changes, glossitis and hair loss, and this is due to its essential role in pathways for growth, reproductive function, immunity as well as neuro-behavioural development [7]. Therefore, bariatric guidelines recommend a multivitamin and mineral supplementation with at least 8–15 mg Zn for every 1 mg Cu following bariatric surgery [5].

A systematic review (SR) on prevalence of Zn deficiency following RYGB reported that 23.9% (351) were diagnosed with asymptomatic Zn deficiency at a mean follow-up of 29.5 months. There were 13 studies included in this SR (*n* = 1469), and the range of Zn deficiency was from 6.35 to 68.0%. The highest rate of 68.0% was seen in the patients who were not given any Zn supplement [26]. This study found only 6 case reports of symptomatic Zn deficiency, with the major presenting clinical symptom being skin rash. The adherence to supplementation was either poor or not available. However, most cases were treated and responded to an oral Zn supplementation. The potential type 2 error and publication bias possibly resulting in underreporting were identified to be the major limitations of their study.

### Copper Abnormalities

The American Society for Metabolic and bariatric Surgery (ASMBS) guidelines [5, 6] recommend the routine screening of Cu through serum Cu and ceruloplasmin for patients prior to RYGB and BPD procedures. However, the British Obesity and Metabolic Surgery Society (BOMSS) did not identify strong evidence to support routine screening of Cu levels [7].

In our study, pre operatively, low Cu level was only seen in 1 (0.7%) patient. This is consistent with a study by Papamargaritis et al. showing minimal level of Cu abnormality (2%) [12]; however, the result contrasts with other findings where Cu deficiency was reported to be 63.8% [28] to as high as 74% [6]. This may represent the different availability of micronutrients in the diets in different countries and potentially in different communities within countries.

On the other hand, elevated Cu levels were more common in this study and were present in 23 (16.7%) patients. In interpreting Cu levels, it is important to note that inflammation affects both serum copper and ceruloplasmin, and hence, the results need to be interpreted with caution. Copper is a positive acute phase reactant [14], and hence, as CRP was elevated in 52 (47.7%) cases, an elevated Cu in this cohort is expected. As observed in this study, the CRP improved following surgery and, hence, so does the Cu levels.

Reports on the prevalence of Cu deficiency following surgery remain inconsistent. However, several studies have reported that Cu deficiency, if untreated, can lead to serious and irreversible neurological complication [10, 12] Therefore, monitoring of the Cu status of bariatric patients is recommended by most bariatric guidelines.

Following surgery in this cohort of patients’ Cu abnormality was negligible with only one patient exhibiting Cu abnormality in the ≥ 2 years post op period. This is consistent with other studies where Cu deficiency was only observed in 0–5% of bariatric patients, 3–36 months following surgery [12].

An SR after RYGB found 5 studies reporting asymptomatic deficiency of Cu with prevalence of 9.38% (23 patients) of the cases [10]. They however also found 17 studies presenting on 34 cases of symptomatic Cu deficiency, which included neurological and haematological symptoms. However, only one patient reported to be adhering to supplementation. These findings reinforce the need for close- and long-term monitoring of biochemistry as well as adherence to supplementation of the bariatric surgical patient.

Another SR on Cu deficiency and the impact of anaemia following bariatric surgery found limited studies reporting on Cu levels, with only few studies showing an increasing deficiency of Cu following surgery [23]. However, they identified the limited high-quality studies in this area and highlight the studies’ inadequate consideration of other contributing factors such as inflammatory markers, adherence to supplementations and quality of the supplements. In our study, we reviewed the CRP as a marker of inflammation and considered the adherence to supplementation for a more comprehensive assessment.

In this study, the case with Cu abnormality was a male patient with a revisional RYGB for treatment of his severe reflux and hiatus hernia following his primary LAGB procedure. He exhibited significant gastrointestinal symptoms such as dumping syndrome, food intolerances as well as unusually diarrhoea and steatorrhoea. Despite reportedly adhering to a multivitamin, iron and vitamin B12 supplementations, he also had abnormalities in Zn, ferritin, B12, active B12, folate, vitamin D, iPTH and total protein. His CRP levels were within normal range. The contributing factors to these additional nutritional deficiencies would be his potentially poor diet tolerance and the reported GI and malabsorptive symptoms. Hence, oral supplementation would not have been adequate to meet his requirements, hence requiring intravenous or intramuscular supplementation to prevent low levels. He ultimately needed a revision of his RYGB, with subsequent resolution of his GI symptoms.

### Combined Zn and Cu Abnormalities

Zinc and copper abnormality and deficiencies have been reported following all bariatric surgical procedures. Both Zn and Cu require the gastric acid environment for digestion and are subsequently absorbed in the proximal small intestine. Following bariatric surgery, the changes in anatomy and physiology result in a reduction of consumption of dietary sources of Zn and Cu as well as impacting the absorption of these trace elements [12, 27, 29]. Furthermore, inadequate Zn and Cu levels in the standard multivitamin supplements, as well as non-adherence to MVS, may further exacerbate this complication.

Change in Zn and Cu values over time has not been reported by many authors. In this study, we found that the mean level of Cu tends to decline over time; however, Zn levels were stable. As mentioned previously, the decline in Cu levels may be as a result of reducing CRP levels and resolution of the pro-inflammatory obesity state following bariatric surgery. Others have found stable serum Cu and Zn concentrations during the first years after bariatric surgery [12].

The relationship between Zn and Cu has also been highlighted in the literature. The evidence shows that excess Zn results in upregulation of metallothionine, which has a higher affinity for Cu, and, hence, prevents its release into plasma, resulting in a Zn-induced Cu abnormality [13]. Hence for every 8–15 mg of Zn, 1 mg Cu supplementation is recommended [5, 7]. Close attention is however needed to the supplementation formulas as not all contain the appropriate quantities and ratios.

### Multivitamin and Mineral Supplements

In our study, comprehensive bariatric multivitamin and mineral formulas, including Cu levels of 0.75 to 3 mg and Zn levels of 15 to 28 mg, were recommended. However, if a patient did not tolerate these, a BD comprehensive over-the-counter formula containing Cu levels of 1.8 mg and Zn levels of 16 mg was recommended. This is slightly below the recommendations of 2 mg of Cu and 30 mg of Zn; however, dietary sources may be complementing these levels, contributing to minimal observed deficiencies. In interpreting the literature, other confounding factors such as inflammation, appropriateness of sample collection in order to avoid contamination, using a metal-free blood collection tube [29], losing patients to follow up, recall bias, as well as underreporting of problems should also be considered. Overall, differences in bariatric procedure, presence of GI symptoms, adequacy of diet, adherence to adequate and appropriate supplementation and appropriateness of sample collection and analysis explain some of the inconsistencies found in the literature, with relation to Zn and Cu abnormalities.

Based on recommendations from the guidelines [5, 7], multivitamin and mineral supplementations were routinely recommended in this study. However, patients were given individualised advice on the formulas to take. This was based on their reported dietary intake, clinical biochemistry and as well as their supplement preferences and tolerance levels. In this cohort, 90% of patients reported to be taking MVS; however, this did reduce to 77% beyond 2 years post-surgery [17]. The literature highlights the poor long-term adherence to the MVS [8, 30]. A general negative attitude to adherence, forgetfulness, cost, GI side effects and intolerance to taste and smell were some of the reported barriers affecting adherence to MVS following bariatric surgery [31]. Hence, reviewing the MVS at each consult and individually considering patients’ challenges, in order to optimise adherence to these is highly recommended.

### GI Symptoms

In this cohort of patients, common GI symptoms reported following surgery were reflux, food intolerances, nausea, vomiting and diarrhoea, the details of which have been prescribed elsewhere [17]. These are consistent with the reported literature [32, 33]. As the gastrointestinal system evolves and also patients cognitively adapt to their new GI capacity, the symptoms frequently resolved over time. However, as evident in this study, those with persistent symptoms are more likely to exhibit the less common abnormalities such as Zn and Cu following surgery. This if untreated, in addition to poor uptake of supplementation, may result in severe complications; some of which are irreversible.

The limitations of our study were those related to the retrospective nature of the study, missing data as well as relying on reported data. Secondly, the majority of patients had SG procedure; hence, the results may not translate to other bariatric procedures. Thirdly, the number of lost to follow ups and, hence, missing data may contribute to the chance of under reporting Cu and or Zn abnormalities in this cohort. Finally, the timing for taking the supplementations as well as dietary intake of Cu and Zn was not considered in this study. These may impact absorption or total intake of Cu and Zn, and hence, the authors recommend considering this for the future studies.

However, with limited studies published on Cu and Zn levels, especially in the Australian population, this study presents a realistic observation of private bariatric clinics and, hence, encourages others to reflect on clinical practice, especially given that most bariatric surgery is conducted privately in Australia [34].

## Conclusion

Bariatric surgery results in durable weight loss; however, nutritional concerns remain an issue. In the initial stages post op, with improvement in inflammatory markers and good adherence to MVS, deficiencies may not be a concern. However, as nutrient stores deplete, patient follow up and the adherence to MVS decline over time and the risk of complications increases. Furthermore, patients with GI symptoms are at higher risk of the less common nutritional abnormalities such as Zn and Cu. These abnormalities may be underreported, as they not routinely screened. The majority are subclinical and may not manifest as harmful side effects. However, for those who may be at risk, due to poor adherence to supplements, gastrointestinal symptoms and malabsorption, potentially irreversible complications from deficiencies may arise. Undertaking a broad blood investigative panel including the less commonly tested micronutrients in these circumstances may optimise treatment and prevent such complications.
